# Clustering of time-course gene expression profiles using normal mixture models with autoregressive random effects

**DOI:** 10.1186/1471-2105-13-300

**Published:** 2012-11-14

**Authors:** Kui Wang, Shu Kay Ng, Geoffrey J McLachlan

**Affiliations:** 1 Department of Mathematics, University of Queensland, Brisbane, QLD 4072, Australia; 2 School of Medicine, Griffith Health Institute, Griffith University, Meadowbrook, QLD 4131, Australia

**Keywords:** Time-course data, Mixtures of linear mixed models, Autoregressive random effects, EMMIX-WIRE procedure

## Abstract

**Background:**

Time-course gene expression data such as yeast cell cycle data may be periodically expressed. To cluster such data, currently used Fourier series approximations of periodic gene expressions have been found not to be sufficiently adequate to model the complexity of the time-course data, partly due to their ignoring the dependence between the expression measurements over time and the correlation among gene expression profiles. We further investigate the advantages and limitations of available models in the literature and propose a new mixture model with autoregressive random effects of the first order for the clustering of time-course gene-expression profiles. Some simulations and real examples are given to demonstrate the usefulness of the proposed models.

**Results:**

We illustrate the applicability of our new model using synthetic and real time-course datasets. We show that our model outperforms existing models to provide more reliable and robust clustering of time-course data. Our model provides superior results when genetic profiles are correlated. It also gives comparable results when the correlation between the gene profiles is weak. In the applications to real time-course data, relevant clusters of coregulated genes are obtained, which are supported by gene-function annotation databases.

**Conclusions:**

Our new model under our extension of the EMMIX-WIRE procedure is more reliable and robust for clustering time-course data because it adopts a random effects model that allows for the correlation among observations at different time points. It postulates gene-specific random effects with an autocorrelation variance structure that models coregulation within the clusters. The developed R package is flexible in its specification of the random effects through user-input parameters that enables improved modelling and consequent clustering of time-course data.

## Background

DNA microarray analysis has emerged as a leading technology to enhance our understanding of gene regulation and function in cellular mechanism controls on a genomic scale. This technology has advanced to unravel the genetic machinery of biological rhythms by collecting massive gene-expression data in a time course. Time-course gene expression data such as yeast cell cycle data [1] appear to be periodically expressed. To associate the profile of gene expression with a physiological function of interest, it is crucial to cluster the types of gene expression on the basis of their periodic patterns. The identification of co-expressed genes also facilitates the prediction of response to treatment or toxic compounds [2]. Statistical modelling and algorithms play a central role in cataloguing dynamic gene-expression profiles.

Various computational models have been developed for gene clustering based on cross-sectional microarray data [3-5]. Also, considerable attention has been paid to methodological derivations for detecting temporal patterns of gene expression in a time course based on functional principal component analysis or mixture model analysis [6-15], including the applications to identify differentially expressed genes over time [16,17].

Finite mixture models [18] have been widely used to model the distributions of a variety of random phenomena. Multivariate normality is generally assumed for multivariate data of a continuous nature. The multivariate normal mixture model is employed to detect different patterns in gene-expression profiles. However, when the two assumptions that are commonly adopted in practice, namely, 

(1) there are no replications on any particular entity specifically identified as such and

(2) all the observations on the entities are independent of one another,

are violated, multivariate normal mixture models may not be adequate. For example, condition (2) will not hold for the clustering of gene profiles, since not all the genes are independently distributed, and condition (1) will generally not hold either as the gene profiles may be measured over time or on technical replicates. While this correlated structure can be incorporated into the normal mixture model by appropriate specification of the component-covariance matrices, it is difficult to fit the model under such specifications. For example, the M-step may not exist in closed form [19].

Accordingly, Ng et al. [13] have developed the procedure called EMMIX-WIRE (^2^**EM**-based **MIX**ture analysis **Wi**th **R**andom Effects) to handle the clustering of correlated data that may be replicated. They adopted a mixture of linear mixed models to specify the correlation structure between the variables and to allow for correlations among the observations. It also enables covariate information to be incorporated into the clustering process [13]. Proceeding conditionally on the tissue-specific random effects as formulated in [13], the E- and M-steps can be implemented in closed form. In particular, an approximation to the E-step by carrying out time-consuming Monte Carlo methods is not required. A probabilistic or an outright clustering of the genes into g components can be obtained, based on the estimated posterior probabilities of component membership given the profile vectors and the estimated tissue-specific random effects; see [13].

Fourier series approximations have been used to model periodic gene expression, leading to the detection of periodic signals in various organisms including yeast and human cells [1,20,21]. If the genes studied are periodically regulated, their time-dependent expression can be accurately approximated by a Fourier series approximation [20]. A general form of the *k*th order Fourier series expansion is given as

(1) $$\begin{array}{c} g_{k}(t)=a_{0}+\overset{k}{\underset{j=1}{\sum }}[a_{j}\text{cos}(2\mathrm{\Pi jt}/\omega )+b_{j}\text{sin}(2\mathrm{\Pi jt}/\omega ], \end{array}$$

where *a*_0_ is the average value of *g*_*k*_(*t*). The other coefficients *a*_*k*_and *b*_*k*_ are the amplitude coefficients that determine the times at which the gene achieves peak and trough expression levels, respectively, and *ω* is the period of the signal of gene expression. While the time-dependent expression value of a gene can be adequately modelled by a Fourier series approximation of the first three orders [14], recent results [13,14] demonstrate that the first-order Fourier series approximation is sufficient to provide good results in terms of clustering the time-course data into meaningful functional groups. Alternatively, the likelihood ratio test may be used to determine the order of the Fourier series approximation within the nested regression models.

The EMMIX-WIRE procedure of Ng et al. [13] is developed primarily for clustering genes from general microarray experimental designs. On the other hand, Kim et al. [14] focus specifically on clustering periodic gene profiles and propose a special covariance structure to incorporate the correlation between observations at different time points. They also review current methods and compare their method with that of Ng et al. [13]. More recently, Scharl et al. [22] use integrated autoregressive (AR) models to create cluster centers in their simulation study of mixtures of regression models for time-course gene expression data through the new version of software FlexMix in Leisch [23]. Wang and Fan [24] propose mixtures of multivariate linear mixed models with autoregressive errors to analyse longitudinal data. In this paper, we propose a new EMMIX-WIRE normal mixture regression model with AR(1) random effects for the clustering of time-course data. In particular, the model accounts for the correlation among gene profiles and models the dependence between expressions over time via AR(1) random effects.

The paper is organized as follow: we first present the development of the extension of the EMMIX-WIRE model to incorporate AR(1) random effects which are fitted under the EM framework. Then in the following section, we conduct a simulation study and the data analysis with three real yeast cell datasets. In the last section some discussion is provided. The technical details of the derivations are provided in the Additional file 1.

## Methods

### EMMIX-WIRE Model with AR(1) Random Effects

We let *X* denote the design matrix and *β*the associated vector of regression coefficients for the fixed effects. In the specification of the mixture of mixed linear components as adopted by Ng et al. [13], the vector ***y***_*j*_ for the *j*th gene conditional on its membership of the *h*th component of the mixture is expressed as

(2) $$y_{j}=X\beta_{h}+Z_{1}u_{\mathrm{jh}}+Z_{2}v_{h}+\varepsilon_{\mathrm{jh}}\;(j=1,\ldots ,n),$$

where *β*_*h*_ is a (2*k* + 1) vector containing unknown parameters *a*_0_*a*_1_,…,*a*_*k*_*b*_1_,…,*b*_*k*_; see (1), *u*_*jh*_=(*u*_*jh*1_,…,*u*_*jhm*_)^*T*^and *v*_*h*_=(*v*_*h*1_,…,*v*_*hm*_)^*T*^are the random effects, where *m* is the number of time points. In (2), *Z*_1_ and *Z*_2_ are *m*×*m* identity matrices. Without loss of generality, we assume *ε*_*jh*_and *v*_*h*_ to be independent and normally distributed, *N*(0,*Ω*) and *N*(0,*D*), independent of *u*_*jh*_. To further account for the time dependent random gene effects, a first-order autoregressive correlation structure is adopted for the gene profiles, so that *u*_*jh*_follows a *N*(0,*θ*^2^*A*(*ρ*)) distribution, where

(3) $$A(\rho )=\frac{1}{1-\rho^{2}}\left(\begin{array}{llll} \;\;1\;\; & \;\;\;\rho & \;\;\ldots & \;\rho^{m-1}\\ \;\;\rho & \;\;\;1 & \;\;\ldots \;\; & \;\rho^{m-2}\\ \vdots \;\; & \;\;\vdots & \;\vdots & \;\;\vdots \\ \rho^{m-1}\;\; & \;\rho^{m-2}\;\; & \;\;\ldots \;\; & \;\;1 \end{array}\right).$$

The inverse of *A*(*ρ*) can be expressed as

(4) $$A{\left(\rho \right)}^{-1}=(1+\rho^{2})I-\mathrm{\rho J}-\rho^{2}K,$$

and

(5) $$\text{trace}\left(\frac{\mathrm{\partial A}{\left(\rho \right)}^{-1}}{\partial \rho }A\left(\rho \right)\right)=-2\rho /(1-\rho^{2}),$$

where *I*, *J*, and *K* are all *m*×*m*matrices. Specifically, *I* is the identity matrix; *J* has its sub-diagonal entries ones and zeros elsewhere, and *K* takes on the value 1 at the first and last element of its principal diagonal and zeros elsewhere. The expressions (4) and (5) are needed in the derivation of the maximum likelihood estimates of the parameters.

The assumptions (2) and (3) imply that our new model assumes an autocorrelation covariance structure under which measurements at each time point have a larger variance compared to the model of Kim et al. [14] under an AR(1) autocorrelation residual structure.

In the context of mixture models, we consider the *g*-component mixture with probability density function (pdf) as

(6) $$f(y|\Psi )=\overset{g}{\underset{h=1}{\sum }}p_{h}f_{h}(y_{j}|\beta_{h},\Omega_{h},\theta_{h}^{2},A_{h},D_{h}),$$

where *f*_*h*_is the component-pdf of the multivariate normal distribution with mean vector *X*_*h*_*β*_*h*_ and covariance matrix

(7) $$\theta_{h}^{2}Z_{1}A_{h}Z_{1}^{T}+Z_{2}D_{h}Z_{2}^{T}+\Omega_{h}.$$

The vector of unknown parameters is denoted by *Ψ*and can be estimated by maximum likelihood via the EM algorithm.

### Maximum likelihood via the EM algorithm

In the EM framework adopted here, the observed data vector *y*=(*y*_1_,*y*_2_,…,*y*_*n*_)^*T*^ is augmented by the unobservable component labels, *z*_1_,*z*_2_,…,*z*_*n*_of *y*_1_,*y*_2_,…,*y*_*n*_, where *z*_*j*_ is the *g*-dimensional vector with *h*th element *z*_*jh*_, which is equal to 1 if *y*_*j*_comes from the *h*th component of the mixture, and is zero otherwise. These unobservable values are considered to be missing data and are included in the so-called complete-data vector. Finally, we take the random effect vectors *u*_*jh*_ and *v*_*h*_(*j*=1,…,*n*; *h*=1,…,*g*), to be missing and include them too in the complete-data vector. Now the so-called complete-data log-likelihood *l*_*c*_ is the sum of four terms *l*_*c*_=*l*_1_ + *l*_2_ + *l*_3_ + *l*_4_, where

(8) $$l_{1}=\overset{g}{\underset{h=1}{\sum }}\overset{n}{\underset{j=1}{\sum }}z_{\mathrm{jh}}\log(p_{h})$$

is the logarithm of the probability of the component labels *z*_*jh*_, and where *l*_2_ is the logarithm of the density function of *y* conditional on *u*_*jh*_,*v*_*h*_, and *z*_*jh*_=1, and *l*_3_ and *l*_4_is the logarithm of the density function of *u* and *v*, respectively, given *z*_*jh*_=1,

(9) $$l_{2}=-\frac{1}{2}\overset{g}{\underset{h=1}{\sum }}\overset{n}{\underset{j=1}{\sum }}z_{\mathrm{jh}}\left(m\log\left(2,\Pi \right)+\log|\Omega_{h}|+\varepsilon_{\mathrm{jh}}^{T}\Omega_{h}^{-1}\varepsilon_{\mathrm{jh}}\right),$$

(10) $$\begin{array}{c} l_{3}=-\frac{1}{2}\overset{g}{\underset{h=1}{\sum }}\overset{n}{\underset{j=1}{\sum }}z_{\mathrm{jh}}\;\left(m\log\left(2,\Pi ,\overset{2}{\underset{h}{\theta }}\right)+\log|A_{h}|+\theta_{h}^{-2}u_{\mathrm{jh}}^{T}A_{h}^{-1}u_{\mathrm{jh}}\right), \end{array}$$

(11) $$l_{4}=-\frac{1}{2}\overset{g}{\underset{h=1}{\sum }}(\overset{n}{\underset{j=1}{\sum }}z_{\mathrm{jh}})\left(m\log\left(2,\Pi \right)+\log|D_{h}|+v_{h}^{T}D_{h}^{-1}v_{h}\right),$$

where

(12) $$\varepsilon_{\mathrm{jh}}=y_{j}-X\beta_{h}-Z_{1}u_{\mathrm{jh}}-Z_{2}v_{h}.$$

To maximize the complete-data log likelihood *l*_*c*_, the above decomposition implies that each of *l*_1_,*l*_2_,*l*_3_, and *l*_4_ can be maximized separately. The EM algorithm proceeds iteratively until the difference between successive values of the log likelihood is less than some specified threshold. All major derivations are given in the Additional file 1.

## Results

### Simulation study

To illustrate the performance of the proposed model, we present a simulation study based on synthetic time-course data. In the following simulation, we consider an autocorrelation dependence for the periodic expressions and compare our model to that of Kim et al. [14]. Synthetic time-course data from three different parametric models (the full model under our new extended EMMIX-WIRE approach denoted by EM-W in the tables, the extended model of Qin and Self [6], and the model of Kim et al. [14]), assuming a first-order Fourier series of periodicity, are considered in the simulation study. Within each model, we consider two different settings of *θ*^2^corresponding to low and high autocorrelation among the periodic gene expressions. We also assume that *Ω*and *D* are diagonal matrices, where the common diagonal elements are represented by *σ*^2^ and *d*^2^, respectively.

There are three clusters of genes. The periods for each cluster are 6, 10, and 16, respectively. There are 24 measurements at time points 0, 1, …, 23, and the first order Fourier expansion is adopted in the simulation models. Parameters and simulation results are listed in Tables 1, 2, 3, 4, 5, 6. In each table, we summarize the results from 1000 simulated sets of data. The true values of the parameters and the biases of their estimates are given in these tables, along with the root mean square errors (RMSEs) in parentheses. We terminated the EM algorithm iterations when the absolute values of the relative changes in all estimates between consecutive iterations were smaller than 0*.*00001, with the maximum iteration of 1000. For our model, we started from the true partition; for the model of Kim et al. [14], we started from the true values of the parameters. Alternatively, initialization procedures have been considered for mixtures of regression models with and without random effects [22]. For the comparison, we consider the misclassified error rate, the Rand Index, and the adjusted Rand Index [25], where the latter two assess the degree of agreement between the partition and the true clusters of genes. A larger (adjusted) Rand Index indicates a higher level of agreement.

**Table 1:** **Bias and RMSE in brackets from 1000 simulated datasets (generated from new EMMIX-WIRE (EM-W) model with **$\theta_{h}^{2}$** equal to 0.5)**

	**First component**		**Second component**		**Third component**	
**Parameters**	**EM-W**	**Kim**	**EM-W**	**Kim**	**EM-W**	**Kim**
*p*(0.585,	-0.002	0.016	-0.009	-0.001	0.011	-0.015
0.1,0.315)	(0.045)	(0.052)	(0.033)	(0.029)	(0.051)	(0.051)
*a*_0_(0.3,	0.002	0.008	-0.006	-0.036	-0.003	-0.009
1,0.2)	(0.135)	(0.137)	(0.175)	(0.186)	(0.186)	(0.182)
*a*_1_(0.03,	-0.001	-0.018	0.024	0.004	0.004	-0.001
1,0.02)	(0.119)	(0.124)	(0.272)	(0.160)	(0.175)	(0.152)
*b*_1_(0.06,	0.009	-0.015	-0.164	0.031	0.027	0.008
0.9,0.01)	(0.119)	(0.132)	(0.223)	(0.160)	(0.149)	(0.183)
*θ*^2^(0.5,	0.055	1.543	0.089	1.346	0.110	1.443
0.5,0.5)	(0.082)	(1.547)	(0.164)	(1.349)	(0.152)	(1.446)
*ρ*(0*.*6	-0.023	-0.395	-0.043	-0.372	-0.043	-0.392
0.6,0.6)	(0.036)	(0.397)	(0.082)	(0.374)	(0.058)	(0.394)
*σ*^2^(1.0,	0.0171		-0.017		0.011	
1.0,1.0)	(0.055)		(0.127)		(0.088)	
*d*^2^(0.4,	-0.112		-0.091		-0.118	
0.2,0.3)	(0.145)		(0.102)		(0.134)	
	EM-W		Kim		Proportion	
	Mean (RMSE) SD		Mean (RMSE) SD		(EM-W is better)	
Error rate	0.036 (0.044) 0.026		0.099 (0.108) 0.044		986/1000	
Rand	0.954 (0.056) 0.032		0.863 (0.149) 0.060		993/1000	
Adjusted	0.906 (0.113) 0.064		0.726 (0.299) 0.120		993/1000	

**Table 2:** **Bias and RMSE in brackets from 1000 simulated datasets (generated from new EMMIX-WIRE (EM-W) model with **$\theta_{h}^{2}$** equal to 1.3**

	**First component**		**Second component**		**Third component**	
**Parameters**	**EM-W**	**Kim**	**EM-W**	**Kim**	**EM-W**	**Kim**
*p*(0.585,	-0.006	0.035	-0.009	-0.002	0.015	-0.033
0.1,0.315)	(0.061)	(0.080)	(0.047)	(0.045)	(0.070)	(0.074)
*a*_0_(0.3,	0.001	0.018	-0.004	-0.069	-0.00	-0.014
1,0.2)	(0.137)	(0.147)	(0.173)	(0.197)	(0.186)	(0.178)
*a*_1_(0.03,	0.010	-0.062	0.017	-0.031	0.001	-0.002
1,0.02)	(0.162)	(0.227)	(0.388)	(0.236)	(0.230)	(0.199)
*b*_1_(0.06,	0.009	-0.042	-0.180	0.073	0.032	0.009
0.9,0.01)	(0.124)	(0.166)	(0.235)	(0.188)	(0.163)	(0.213)
*θ*^2^(1.3,	-0.042	1.671	-0.030	1.449	0.008	1.549
1.3,1.3)	(0.097)	(1.677)	(0.223)	(1.460)	(0.153)	(1.556)
*ρ*(0.6	0.009	-0.249	-0.001	-0.228	0.002	-0.250
0.6,0.6)	(0.020)	(0.251)	(0.055)	(0.235)	(0.025)	(0.252)
*σ*^2^(1.0,	0.131		0.121		0.141	
1.0,1.0)	(0.155)		(0.219)		(0.186)	
*d*^2^(0.4,	-0.151		-0.124		-0.160	
0.2,0.3)	(0.172)		(0.129)		(0.168)	
	EM-W		Kim		Proportion	
	Mean (RMSE) SD		Mean (RMSE) SD		(EM-W is better)	
Error rate	0.094 (0.102) 0.039		0.184 (0.192) 0.053		988/1000	
Rand	0.881 (0.129) 0.049		0.758 (0.252) 0.069		1000/1000	
Adjusted	0.760 (0.259) 0.097		0.518 (0.500) 0.133		1000/1000	

**Table 3:** **Bias and RMSE in brackets from 1000 simlated datasets (generated from new EMMIX-WIRE (EM-W) model with **$\theta_{h}^{2}$** equal to 0.5 and *d*^2 ^equal to 0)**

	**First component**		**Second component**		**Third component**	
**Parameters**	**EM-W**	**Kim**	**EM-W**	**Kim**	**EM-W**	**Kim**
*p*(0.585,	0.001	0.008	-0.001	-0.003	-0.001	-0.005
0.1,0.315)	(0.009)	(0.012)	(0.008)	(0.008)	(0.010)	(0.011)
*a*_0_(0.3,	0.001	0.008	-0.001	-0.018	0.003	-0.014
1,0.2)	(0.017)	(0.019)	(0.018)	(0.026)	(0.016)	(0.016)
*a*_1_(0.03,	-0.002	-0.023	-0.001	-0.005	0.003	-0.006
1,0.02)	(0.049)	(0.060)	(0.059)	(0.062)	(0.049)	(0.049)
*b*_1_(0.06,	-0.001	-0.014	0.016	0.019	0.002	0.004
0.9,0.01)	(0.026)	(0.031)	(0.033)	(0.038)	(0.032)	(0.033)
*θ*^2^(0.5,	0.071	1.162	0.081	1.158	0.078	1.159
0.5,0.5)	(0.081)	(1.162)	(0.119)	(1.160)	(0.090)	(1.159)
*ρ*(0.6	-0.032	-0.337	-0.037	-0.339	-0.036	-0.339
0.6,0.6)	(0.038)	(0.337)	(0.062)	(0.340)	(0.045)	(0.340)
*σ*^2^(1.0,	-0.059		-0.069		-0.064	
1.0,1.0)	(0.068)		(0.106)		(0.077)	
*d*^2^(0,	0		0.001		0.000	
0,0)	(0.000)		(0.001)		(0.001)	
	EM-W		Kim		Proportion	
	Mean (RMSE) SD		Mean (RMSE) SD		(EM-W is better)	
Error rate	0.078 (0.078) 0.008		0.081 (0.081) 0.009		738/1000	
Rand	0.891 (0.110) 0.012		0.886 (0.115) 0.012		806/1000	
Adjusted	0.780 (0.222) 0.023		0.769 (0.232) 0.025		802/1000	

Specifically, we first investigate the performance of our new extended EMMIX-WIRE model and that of Kim et al. [14] when the data are generated from the extended EMMIX-WIRE model, in which gene expressions within a cluster are correlated. As listed in Tables 1 and 2, the estimates of the parameters *pa*_0_*a*_1_*b*_1_*θ*^2^*ρ*, and *σ*^2^ in the proposed model are approximately unbiased, except for *d*^2^, which is slightly underestimated. In contrast, the model of Kim et al. [14] fails to capture the contributions from gene-specific and tissue-specific effects on the autocorrelation among periodic gene expressions at each time point, and thus overestimates the correlation between different time points for each gene. Their method therefore leads to an inferior clustering performance in terms of higher error rates and smaller Rand Indices. From Tables 1 and 2, our proposed method performs better in more than 98% out of 1000 simulated datasets. It also has smaller RMSEs (relative to 0 for error rates and to 1 for Rand Indices) and the difference in performance is significant based on the standard deviation (SD) of the error rates and Rand Indices.

We now compare our model with that of Kim et al. [14], using the data from the extended model of Qin and Self [6], which is a special case of our EMMIX-WIRE model (with *d*^2^ = 0), where gene expressions are independent. The results are presented in Tables 3 and 4, where it can be seen that our method provides essentially unbiased estimates of all the parameters. On the other hand, the model of Kim et al. [14] still overestimates the residual variance at different time points and underestimates the correlation between different time points for each gene, as it fails to capture the contribution from gene-specific effects to the autocorrelation among periodic gene expressions at each time point. Their method again produces slightly larger error rates and smaller Rand Indices, though the difference is not significant. From Tables 3 and 4, the proposed method indeed performs better with slightly larger Rand Indices in more than 80% of 1000 simulated datasets.

**Table 4:** **Bias and RMSE in brackets from 100 simulated datasets (generated from new EMMIX-WIRE (EM-W) model with **$\theta_{h}^{2}$** equal to 1.3 and *d*^2 ^equal to 0)**

	**First component**		**Second component**		**Third component**	
**Parameters**	**EM-W**	**Kim**	**EM-W**	**Kim**	**EM-W**	**Kim**
*p*(0.585,	-0.001	0.024	0.002	-0.005	-0.001	-0.019
0.1,0.315)	(0.014)	(0.029)	(0.016)	(0.017)	(0.017)	(0.026)
*a*_0_(0.3,	-0.001	0.018	0.003	-0.046	0.000	-0.005
1,0.2)	(0.027)	(0.035)	(0.026)	(0.053)	(0.021)	(0.021)
*a*_1_(0.03,	0.001	-0.068	0.005	-0.041	0.001	0.008
1,0.02)	(0.085)	(0.146)	(0.108)	(0.127)	(0.086)	(0.085)
*b*_1_(0.06,	0.003	-0.031	0.005	0.047	0.002	0.004
0.9,0.01)	(0.042)	(0.063)	(0.054)	(0.072)	(0.050)	(0.054)
*θ*^2^(1.3,	-0.059	1.254	-0.076	1.251	-0.052	1.242
1.3,1.3)	(0.087)	(1.254)	(0.178)	(1.257)	(0.104)	(1.243)
*ρ*(0.6	0.012	-0.198	-0.013	-0.201	0.009	-0.203
0.6,0.6)	(0.019)	(0.199)	(0.039)	(0.206)	(0.023)	(0.204)
*σ*^2^(1.0,	0.046		0.056		0.039	
1.0,1.0)	(0.070)		(0.145)		(0.084)	
*d*^2^(0.,	0.000		0.001		0.000	
0.,0.)	(0.000)		(0.001)		(0.000)	
	EM-W		Kim		Proportion	
	Mean (RMSE) SD		Mean (RMSE) SD		(EM-W is better)	
Error rate	0.154 (0.154) 0.011		0.161 (0.162) 0.012		835/1000	
Rand	0.796 (0.204) 0.014		0.783 (0.217) 0.016		912/1000	
Adjusted	0.590 (0.411) 0.028		0.566 (0.435) 0.031		896/1000	

Lastly, we generate the data from the model of Kim et al. [14] and provide comparative results in Tables 5 and 6. It is observed from Tables 5 and 6 that the clustering performances are comparable between the two models, as the difference is not significant. Out of 1000 simulated datasets, the proposed method is not worse in more than 35%.

**Table 5:** **Bias and RMSE in brackets from 1000 simulated datasets (generated from **[14]** with **$\theta_{h}^{2}$** equal to 0.5)**

	**First component**		**Second component**		**Third component**	
**Parameters**	**EM-W**	**Kim**	**EM-W**	**Kim**	**EM-W**	**Kim**
*p*(0.585,	-0.003	0.000	-0.008	0.001	0.010	-0.000
0.1,0.315)	(0.004)	(0.003)	(0.023)	(0.003)	(0.024)	(0.004)
*a*_0_(0.3,	0.002	0.000	0.003	0.001	0.001	0.001
1,0.2)	(0.013)	(0.013)	(0.010)	(0.010)	(0.010)	(0.010)
*a*_1_(0.03,	0.015	0.001	-0.236	-0.002	0.047	0.003
1,0.02)	(0.041)	(0.036)	(0.333)	(0.037)	(0.073)	(0.035)
*b*_1_(0.06,	0.014	-0.000	-0.308	-0.001	0.058	0.001
0.9,0.01)	(0.026)	(0.021)	(0.345)	(0.023)	(0.067)	(0.025)
*θ*^2^(0.5,	-0.034	-0.000	-0.006	-0.001	-0.021	-0.000
0.5,0.5)	(0.036)	(0.006)	(0.027)	(0.015)	(0.025)	(0.009)
*ρ*(0.6	0.020	-0.000	0.013	-0.001	0.023	-0.001
0.6,0.6)	(0.021)	(0.007)	(0.025)	(0.017)	(0.028)	(0.009)
*σ*^2^(0.0,	0.025		0.014		0.022	
0.0,0.0)	(0.026)		(0.015)		(0.023)	
*d*^2^(0,	0.000		0.045		0.042	
0,0)	(0.000)		(0.095)		(0.056)	
	EM-W		Kim		Proportion	
	Mean (RMSE) SD		Mean (RMSE) SD		(EM-W is not worse)	
Error rate	0.018 (0.019) 0.006		0.016 (0.017) 0.004		422/1000	
Rand	0.978 (0.023) 0.006		0.980 (0.021) 0.005		365/1000	
Adjusted	0.955 (0.046) 0.012		0.959 (0.042) 0.011		363/1000	

**Table 6:** **Bias and RMSE in brackets from 1000 simulated datasets (generated from **[14]** with **$\theta_{h}^{2}$** equal to 1.3)**

	**First component**		**Second component**		**Third component**	
**Parameters**	**EM-W**	**Kim**	**EM-W**	**Kim**	**EM-W**	**Kim**
*p*(0.585,	-0.009	0.001	-0.007	0.005	0.016	-0.001
0.1,0.315)	(0.013)	(0.010)	(0.012)	(0.011)	(0.020)	(0.013)
*a*_0_(0.3,	-0.002	-0.000	0.015	0.001	0.003	-0.000
1,0.2)	(0.023)	(0.023)	(0.024)	(0.019)	(0.016)	(0.016)
*a*_1_(0.03,	-0.005	-0.001	0.054	-0.000	0.003	0.000
1,0.02)	(0.071)	(0.074)	(0.0928)	(0.083)	(0.068)	(0.064)
*b*_1_(0.06,	0.015	-0.000	-0.131	0.001	0.020	0.000
0.9,0.01)	(0.036)	(0.036)	(0.135)	(0.045)	(0.041)	(0.043)
*θ*^2^(1.3,	-0.195	-0.000	-0.185	-0.003	-0.186	-0.002
1.3,1.3)	(0.196)	(0.016)	(0.192)	(0.049)	(0.189)	(0.025)
*ρ*(0.6	0.043	-0.000	0.037	-0.002	0.044	-0.001
0.6,0.6)	(0.043)	(0.007)	(0.042)	(0.022)	(0.045)	(0.010)
*σ*^2^(0.0,	0.144		0.131		0.143	
0.0,0.0)	(0.145)		(0.133)		(0.144)	
*d*^2^(0.,	0.000		0.000		0.001	
0.,0.)	(0.000)		(0.001)		(0.001)	
	EM-W		Kim		Proportion	
	Mean (RMSE) SD		Mean (RMSE) SD		(EM-W is not worse)	
Error rate	0.103 (0.104) 0.009		0.102 (0.103) 0.010		426/1000	
Rand	0.864 (0.137) 0.012		0.866 (0.135) 0.012		360/1000	
Adjusted	0.725 (0.276) 0.025		0.729 (0.272) 0.025		352/1000	

Our model again provides unbiased estimates for all parameters. In contrast to the model of Kim et al. [14], our model accounts for the correlation among gene profiles via the linear effects modelling. As presented in Tables 1 to 6, our model outperforms the model of Kim et al. [14] when the genetic profiles are correlated. When the genetic profiles are generated independently, our model has slightly better performance in cases where the variability in gene expressions at each time point is large. In cases where the residual covariance structure follows an AR(1) model as in Kim et al. [14], our model still provides comparative results and unbiased estimates as with Kim et al. the model of [14]. The advantage of our model to provide more reliable and robust clustering of time-course data is apparent. With microarray experiments including those time-course studies, gene expression levels measured from the same tissue sample (or time point) are correlated [19], clustering methods which assume independently distributed gene profiles, such as the model of Kim et al. [14], may overlook important sources of variability in the experiments, resulting in the consequent possibility of misleading inferences being made [13].

### Applications: Yeast cell cycle datasets

#### Yeast cell cycle dataset 1

The first example considers the yeast cell cycle data analysed recently by Wong et al. [26]. This dataset (extracted from Cho et al. [27]) is available from Yeung et al. [28]. It contains 237 genes and 17 samples. These genes are categorized with respect to the four categories in the MIPS database (DNA synthesis and replication, organization of centrosome, nitrogen, and sulphur metabolism, and ribosomal proteins). These categories are assumed to represent the true clusters. In this illustration, we fit our new extended EMMIX-WIRE model and the model of Kim et al. [14] to the yeast cell cycle data, with the period of 85 in the Fourier extension [9].

**Figure 1:**
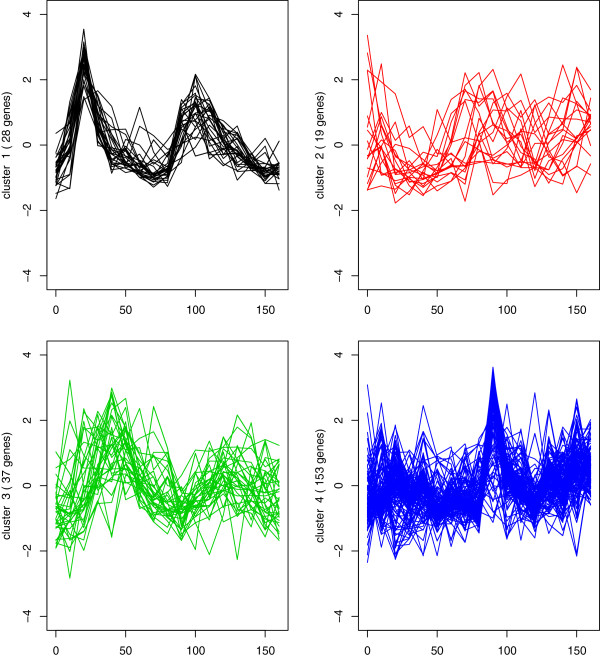
Clustering of gene expression profiles into four groups for the yeast dataset 1.

In Table 2 of Wong et al. [26], it shows that the Rand and adjusted Rand Indices for their two-stage method are 0.7087 and 0.3697, respectively, and these indices are higher than other methods considered in their paper. Using the model of Kim et al. [14], the Rand indices are 0.7330 and 0.4721, respectively. With the EMMIX-WIRE model of Ng et al. [13], we have the Rand and adjusted Rand Indices 0.7799 and 0.5568, respectively. Using the proposed new model, the Rand and adjusted Rand Indices are 0.8123 and 0.6189, respectively, and are the best matches (the largest index) compared with the aforementioned models. The four clusters of genes time-course profiles are presented in Figure 1. It can be seen that the genes have very similar expression patterns within each cluster, except in cluster 2, where there is greater individual variation by some of the genes. The estimation using the proposed model is listed in Table 7. It can be seen that the correlations in the first three components are from 0.27 to 0.72, indicating a significant correlation among gene expressions at different time points. Ignoring this correlation may therefore lead to a lower Rand Index, that is, a worse clustering. We can see the estimates of *d*^2^in clusters 1 and 4 are large and are greater than the corresponding estimates of *θ*^2^, indicating coregulation in these two clusters. If we ignore such within-cluster coregulation, we will have Rand Indices similar to those for the model of Kim et al. [14]. Our model considers both autocorrelation and coregulation, and thus obtains the best clustering performance.

**Table 7:** Estimation of parameters for the yeast cell cycle dataset 1 (237 genes)

	**First cluster**	**Second cluster**	**Third cluster**	**Fourth cluster**
*p*	0.104	0.054	0.118	0.724
*a*_1_	-0.107	0.400	-0.807	0.298
*b*_1_	1.009	-0.119	-0.053	0.079
*σ*^2^	0.027	0.011	0.025	0.278
*θ*^2^	0.174	0.417	0.443	0.307
*ρ*	0.278	0.717	0.435	0.053
*d*^2^	0.191	0.001	0.031	0.310
*ω*	85	85	85	85

#### Yeast cell cycle dataset 2

The second example is the subset of 384 genes from the yeast cell cycle data in Cho et al. [27], corresponding to five functional groups [28].

**Figure 2:**
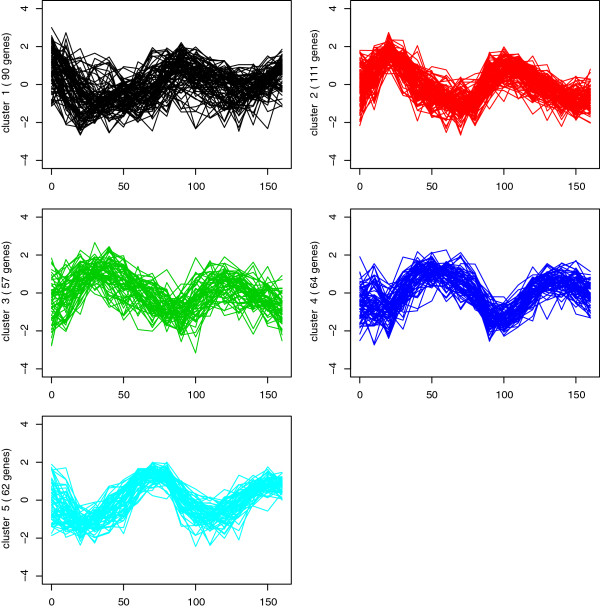
Clustering of gene expression profiles into five groups for the yeast dataset 2.

Each of gene is assigned a “phase”. We call each “phase” a “Main Group”. There are five “Main Groups” in this dataset, namely, early G1, late G1, S, G2, and M. We now compare and assess the cluster quality with the external criterion (the 5 phases). The raw data are log transformed and normalized by columns and rows. Figure 2 presents the five clusters of genes profiles obtained using the proposed model. It can be seen that the genes have very similar expression patterns within each cluster. The estimations are listed in Table 8. The Rand and adjusted Rand Indices are 0.8102 and 0.4484, respectively. They are 0.8108 and 0.4592 for the model of Kim et al. [14]. The error rates are the same (0.2813) for the two models. The performances of the two models are very similar because the correlation among gene profiles is weak in this dataset. As indicated in Table 8, the estimates of *d*^2^ are all very small compared to the estimates of *θ*^2^.

**Table 8:** Estimation of parameters for the yeast cell cycle dataset 2 (384 genes)

	**First cluster**	**Second cluster**	**Third cluster**	**Fourth cluster**	**Fifth cluster**
*p*	0.238	0.290	0.151	0.165	0.157
*a*_1_	0.643	-0.061	-0.736	-0.616	0.329
*b*_1_	-0.062	1.019	0.285	-0.772	-1.001
*σ*^2^	0.011	0.046	0.037	0.028	0.006
*θ*^2^	0.498	0.296	0.470	0.309	0.244
*ρ*	0.503	0.269	0.364	0.379	0.550
*d*^2^	0.062	0.052	0.044	0.065	0.030
*ω*	85	85	85	85	85

#### A complete Yeast dataset

With this third example, we demonstrate how the proposed method can be adopted to cluster a large amount of yeast genes of which only a small proportion shows periodicity. The original dataset consists of more than 6000 genes, where the yeast cells were sampled at 7 min intervals for 119 min with a total of 18 time points after synchronization [20]. By comparing the ‘aggregate’ numerical score (on the basis of a Fourier algorithm for testing periodicity) for each gene relative to a threshold score, 800 genes were identified as periodically regulated [20]. The threshold score was determined empirically, where 91% of previously known cell cycle-regulated genes have a higher score than the threshold. The number of false positives among these 800 cell cycle regulated genes was estimated to be between 3% and 10% [20]. In this study, we worked with 4489 genes that have no missing expression levels across any of the 18 time points. Of these 4489 genes, 612 are periodically regulated and 3877 are not periodic.

**Figure 3:**
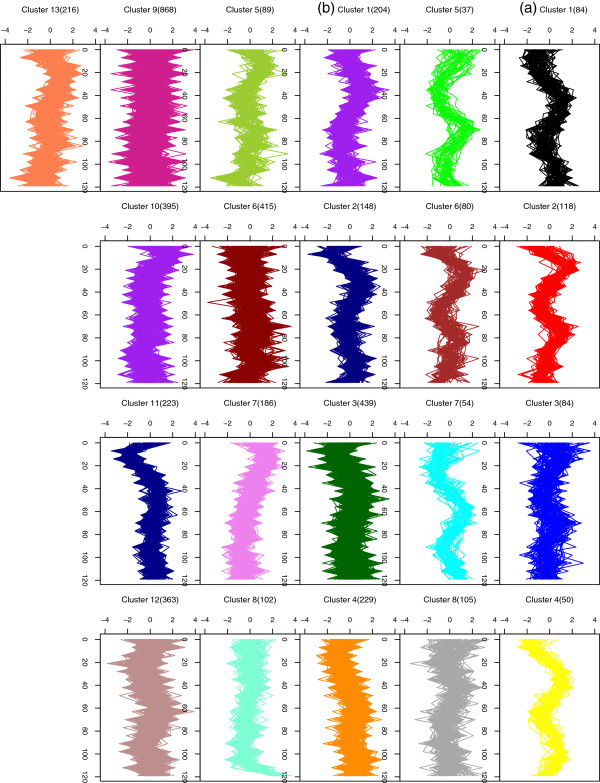
Clustering of gene expression profiles into twenty-one groups for the complete yeast dataset: (a) eight clusters of periodic genes; (b) thirteen clusters of non-periodic genes.

The new mixture model with AR(1) random effects and Fourier series approximations was fitted to the periodic gene expression data with the number of clusters *g*=1 to *g*=20, where the cell cycle period *ω*=63 was determined using a global grid search method described in the Discussion section. The optimal number of clusters was determined using the Bayesian Information Criterion (BIC) of Schwarz [29]. The use of BIC for model selection has been considered in the analysis of gene expression data including [8,13,28]. Based on the BIC, there are eight clusters of periodically regulated genes. With the 3877 non-periodic genes, we adopted the same mixture model with AR(1) random effects, but replacing the Fourier series approximations (1) by a time-series regression form with B-splines [8]. Model selection via BIC indicated that there are thirteen clusters of non-periodic genes. Figure 3 presents the expression profiles of genes in each of the twenty-one clusters. From Figure 3(a), it can be seen that the genes have very similar expression patterns within each cluster, except in clusters 3 and 8, where there is greater individual variation by some of the genes. In Table 9, we give the composition of the eight clusters with respect to the five phases of peak expression. Our clusters 2 and 6 consist mainly of those genes with typical G1 peak expression, while most genes in cluster 1 show G2/M or S/G2 phases of peak expression. On the other hand, a majority of genes in cluster 5 has a typical M/G1 phase and those in cluster 7 have a G2/M phase.

**Table 9:** Distribution of five phases of peak expression over eight clusters obtained (complete yeast data)

**cluster**	**G1**	**G2/M**	**M/G1**	**S**	**S/G2**
1	1	40	0	1	42
2	98	0	19	0	1
3	24	24	31	3	2
4	16	1	0	20	13
5	0	7	30	0	0
6	72	1	3	3	1
7	0	51	1	0	2
8	12	34	8	20	31

With reference to the findings by Spellman et al. [20], a majority of genes in our identified clusters are coregulated. For example, cluster 1 contains genes previously classified to the “CLB2” cluster of Spellman et al. These genes, including ACE2, BUD4, CDC5, and CLB1, are regulated by the MCM1 and SFF transcription factors that induce genes during mitosis [20]. Cluster 8 contain genes described by Spellman et al. as the “MET” cluster. These genes, such as six MET genes and ECM17, are likely to be involved in methionine metabolism [20]. In addition, genes in our clusters 2 and 5 are the major members of the “CLN2” and “SIC1” clusters described in Spellman et al., respectively. The former cluster includes CLN2, CDC9, CDC45, RNR1, POL12, POL30, and are involved in DNA replication and repair. Cluster 5 contains genes that are strongly cell cycle regulated, such as SIC1, TEC1, ASH1, PIR1, and PIR3 [20].

## Discussion

**Figure 4:**
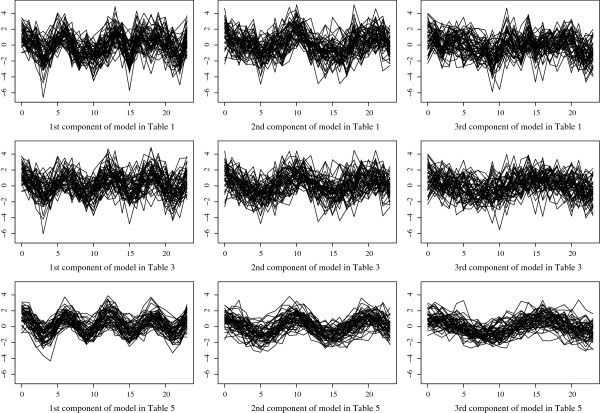
Simulated gene expression profiles for the three models.

We have presented a new mixture model with AR(1) random effects for the clustering of time-course gene expression profiles. Our new model involves three elements taking important role in modelling time-course periodic expression data, namely, (a) Fourier expansion which models the periodic patterns; (b) autocorrelation variance structure that accounts for the autocorrelation among the observations at different time points; and (c) the cluster-specific aandom effects which incorporate the coregulation within the clusters. In particular, the latter two elements corresponding to the correlations between time-points and between genes are crucial for reliable and accurate clustering of time-course data. We have demonstrated in the simulation and real examples that the accuracy of clustering is improved if the autocorrelation among the time dependent gene expression profiles has been accounted for along the time points; this is also demonstrated in Kim et al. [14]. Furthermore, better results are obtained if the coregulation within the clusters is modelled appropriately. To justify whether an autoregressive correlation structure for the gene expression profiles is appropriate, besides reporting the sample correlation as suggested by one of the reviewers, one can also compare the estimated random components and residual variance with each other. When the correlation between genetic profiles is not small, which is the case for typical time-course data, ignorance of this dependency may lead to less accurate clustering results. To further illustrate this, we generated 50 gene expression profiles for each of the three clusters from the three parametric models presented in Tables 1, 3, and 5, respectively. The simulated profiles displayed in Figure 4 highlight the differences between the three models we considered in this paper. The results indicate that the proposed time dependent random gene effects are able to capture different degrees of correlation between gene expression profiles within a cluster. Our method performs better when the degree of correlation is large (Figures 4(a) and 4(b)) and provides comparable results when the degree of correlation is small (Figure 4(c)). This finding is also pronounced in the analyses of the real datasets, where yeast cell cycle dataset 1 (Figure 1) shows a high degree of correlation between profiles and yeast cell cycle dataset 2 (Figure 2) shows a relatively small degree of correlation between profiles.

As an additional empirical comparison, we applied a simple *k*-means clustering procedure to all the simulated and real datasets considered in the paper. We found that the *k*-means procedure gave higher error rate and smaller adjusted Rand index (and both with higher variability), especially when the correlation between genetic profiles is not small. For example, the mean (SE) of the error rate and adjusted Rand index obtained for the *k*-means procedure for the model in Table 1 are 0.062 (0.057) and 0.849 (0.103), rsepectively. For the model in Table 2, they are 0.357 (0.074) and 0.361 (0.103), respectively. With the model in Table 3, they are 0.187 (0.048) and 0.551 (0.077), respectively, while they are 0.464 (0.038) and 0.153 (0.038), respectively, for the model in Table 4. With the models in Tables 5 and 6, where the degree of correlation is small, the mean (SE) of the error rate and adjusted Rand index obtained for the *k*-means procedure are 0.045 (0.018) and 0.876 (0.041), respectively, in Table 5, while they are 0.443 (0.039) and 0.187 (0.041), respectively, in Table 6. For the Yeast 1 real dataset, the adjusted Rand index is 0.509 for the *k*-means procedure, which is smaller than the two methods that are based on the EMMIX-WIRE model. Using the *k*-means procedure, the error rate and adjusted Rand index are 0.404 and 0.442, respectively, for the Yeast 2 dataset. This error rate is the highest among the methods considered in the paper, while the adjusted Rand index is comparable to the other methods. With the complete yeast dataset, the results obtained using the *k*-means procedure are somewhat different from those for our method. For example, we have identified a majority of yeast genes (81%) in cluster 5 which show a typical M/G1 phase, while the cluster obtained by the *k*-means procedure contains only 69% of genes with a M/G1 phase. Moreover, the clusters obtained by the *k*-means procedure for the non-periodic genes are very different from those presented in Figure 3(b) using our method. These findings indicate that a more complex method is generally required for the clustering of time-course data, especially when the correlation between the expression levels is not weak.

For the purpose of comparison, the periods of the signal of gene expression are assumed to be known in the simulation study and applications to real data. In practice, there are several ways to estimate the periods for each cluster [9,13,14,20]. For example, in Kim et al. [14], the periods are estimated using simplex algorithm at the M-step during the EM algorithm. However, when the periods are estimated during the EM iterations, we find that the periods depend also on other parameters. In addition, when we start from an initial period and get the design matrix X, then with higher possibility the best period will be the initial periods. So we change the strategy to a slow one, and we call it global grid search method, which guarantees the highest maximum log likelihood at the best periods. It is implemented as follows. Let *S* be the space with typical element (a vector) (*ω*_1_*ω*_2_, …, *ω*_*g*_)^*T*^, representing the component periods, where *ω*_*h*_can take all possible values (grid points). For example, for the yeast cell cycle data, the possible periods are 60,61, …, 90. Then for each fixed (*ω*_1_*ω*_2_, …, *ω*_*g*_)^*T*^, we estimate the parameters as if the periods for each component were known. Finally, we compare the log likelihood and choose the one with the highest log likelihood as the final result. Since it is very slow if there are too many elements in *S* when we have no prior information about the periods, we recommend using other methods to obtain the periods in such cases, such as the weighted least-squares estimation approach considered in [15]. In all the calculations in this paper, we assume the periods are fixed.

The proposed model is very flexible through the different specifications of design matrices or model options as originally available in Ng et al. [13]. For example, besides the full model, it enables us to incorporate the model of Qin and Self [6] as a special case. Specifically, we can obtain their model by assuming zero cluster effects (*v* = 0) and that random effects *u* be autocorrelated for each gene. Furthermore, when both random effects *u* and *v* are assumed to be zero, then we have normal mixture of regression models. In the program we have developed, there are many options and parameters for users to specify the models they want to use in addition to the models we list in our paper. For example, the developed program is applicable to cluster time-course gene expression profiles that are not periodic (see Figure 3(b)). When periodicity is not obvious, Fourier seris approximations (Equation (1)) can be replaced by a time-series regression form (such as cubic or spline function) to model the conditional mean expression profiles for each component. With reference to Equation (2), the proposed mixture model framework with time dependent AR(1) random gene effects is again desriable to capture the dependence between the expression measurements over time. The program is written in an R package and is available in Additional file 2, which also contains the data.

## Conclusions

Our new extended EMMIX-WIRE model is more reliable and robust for clustering time-course data because it postulates gene-specific random effects with an autocorrelation variance structure that models coregulation within the clusters. The developed R package is flexible in its specification of the random effects through user-input parameters that enables improved modelling and consequent clustering of time-course data.

### Availability

An R-program is available on request from the corresponding author.

## Competing interests

The authors declare that they have no competing interests.

## Authors’ contributions

All authors contributed to the production of the manuscript. SKN and GJM directed the research. KW wrote the R-program and analysed the simulated and real data. All authors read and approved the final manuscript.

## Supplementary Material

Additional file 1 Supplementary file bmcbioinf-supp-2012.pdf Click here for file

Additional file 2 Supplementary file for code and data supp2.zip Click here for file
