# Supplementary material for: Clustering of time-course gene expression profiles using normal mixture models with autoregressive random effects
Source: BMC Bioinformatics. 2012 Nov 14;13:300. doi: 10.1186/1471-2105-13-300 (PMC3574839; doi:10.1186/1471-2105-13-300)
Supplement: Additional file 1 — Supplementary file bmcbioinf-supp-2012.pdf [file 1471-2105-13-300-S1.pdf]

Clustering of time-course gene expression profiles using normal mixture models with autoregressive random effects

Kui Wang[1], Shu Kay Ng[2] and Geoffrey J McLachlan[*1]

[1]Department of Mathematics, University of Queensland, Brisbane, QLD 4072, Australia
[2]School of Medicine, Griffith Health Institute, Griffith University, Meadowbrook, QLD 4131, Australia

Email: K Wang - kwang@maths.uq.edu.au; S K Ng - s.ng@griffith.edu.au; G J McLachlan*- g.mclachlan@uq.edu.au;

*Corresponding author

Joint distribution

Concerning the fitting of the EMMIX-WIRE model in the main text, we assume $\boldsymbol{y}^h = (\boldsymbol{y}_{h1}^T, \ldots, \boldsymbol{y}_{hn_h}^T)^T$ are from the hth cluster, where n_h is the number of observations that belong to the hth cluster ($h = 1, \ldots, g$). The joint distribution of $\boldsymbol{y}^h$ and the random effects u_{jh}, and v_h is given by

$$\begin{bmatrix} u_{jh} \\ v_h \\ \boldsymbol{y}^h \end{bmatrix} \sim N\left(\begin{bmatrix} \mathbf{0} \\ \mathbf{0} \\ W \end{bmatrix}, \begin{bmatrix} \theta_h A_h & \mathbf{0} & V_{13} \\ \mathbf{0}^T & D_h & V_{23} \\ V_{13}^T & V_{23}^T & V_{33} \end{bmatrix} \right), \tag{1}$$

where

$$\begin{aligned} W &= \mathbf{1}_{n_h} \otimes (X_h \beta_h), \\ V_{13} &= \mathbf{1}_{n(j)}^T \otimes (\theta_h A_h Z_1^T), \\ V_{23} &= \mathbf{1}_{n(j)}^T \otimes (D_h Z_2^T), \\ V_{33} &= I_{n_h} \otimes \Sigma_h + J_{n_h} \otimes B_h. \end{aligned}$$

In the above formula, we have $\Sigma_h = \Omega_h + \theta_h Z_1 A_h Z_1^T$, $B_h = Z_2 D_h Z_2^T$, $\mathbf{1}_{n_h}$ is the n_h dimensional vector of one, $\mathbf{1}_{n(j)}$ is the vector with jth element as one and others zeros, and I_{n_h} is the identity matrix and J_{n_h} is the n_h by n_h matrix with all elements as ones. In addition, it can be proved that

$$(I_{n_h} \otimes \Sigma_h + J_{n_h} \otimes B_h)^{-1} = I_{n_h} \otimes \Sigma_h^{-1} - J_{n_h} \otimes C_h,$$

where

$$C_h = \Sigma_h^{-1} B_h (\Sigma_h + n_h B_h)^{-1}$$

and

$$n_h C_h = \Sigma_h^{-1} - (\Sigma_h + n_h B_h)^{-1}.$$

These equations are required in the E-step of the EM algorithm.

The E-step: Conditional expectations

In the EM framework for the proposed mixture model, we have

$$\begin{aligned}
\Sigma_h^{(r)} &= \Omega_h^{(r)} + \theta_h^{(r)} Z_1 A_h^{(r)} Z_1^T, \\
B_h^{(r)} &= Z_2 D_h^{(r)} Z_2^T, \\
M_h^{(r)} &= (\Sigma_h^{(r)} + n_h^{(r)} B_h^{(r)})^{-1}, \\
n_h^{(r)} &= \sum_{i=1}^{n} \tau_{jh}^{(r)}.
\end{aligned}$$

From Ng et al. [1], it follows that the posterior probability τ_{jh} at the rth iteration is given by

$$\begin{aligned}
\tau_{jh}^{(r)} &= P(z_{jh} = 1 | y, \Psi^{(r)}) \\
&= \pi_h^{(r)} f_h(y_j; \Psi^{(r)}) / \sum_{h=1}^{g} \pi_h^{(r)} f_h(y_j; \Psi^{(r)}).
\end{aligned}$$

In the E-step of the EM algorithm, we have to calculate

$$\begin{aligned}
u_{jh}^{(r)} &= E(u_{jh} | y, \Psi^{(r)}) \\
&= \theta_h^{(r)} A_h^{(r)} Z_1^T \Sigma_h^{(r)-1} (y_j - X_h \beta_h^{(r)}) \\
&\quad - \theta_h^{(r)} A_h^{(r)} Z_1^T \Sigma_h^{(r)-1} B_h^{(r)} M_h^{(r)} \sum_{j=1}^{n} \tau_{jh}^{(r)} (y_j - X_h \beta_h^{(r)}), \\
S_{uh}^{(r)} &= \operatorname{cov}(u_{jh} | y, \Psi^{(r)}) \\
&= \theta_h^{(r)} A_h^{(r)} - (\theta_h^{(r)})^2 A_h^{(r)} Z_1^T M_h^{(r)} Z_1 A_h^{(r)} \\
&\quad - (\sum_{j=1}^{n} \tau_{jh}^{(r)} - 1)(\theta_h^{(r)})^2 A_h^{(r)} Z_1^T \Sigma_h^{(r)-1} Z_1 A_h^{(r)}, \\
v_h^{(r)} &= E(v_h | y, \Psi^{(r)}) \\
&= D_h^{(r)} Z_2^T M_h^{(r)} \sum_{j=1}^{n} \tau_{jh}^{(r)} (y_j - X_h \beta_h^{(r)}), \\
S_{vh}^{(r)} &= \operatorname{cov}(v_h | y, \Psi^{(r)}) \\
&= D_h^{(r)} - D_h^{(r)} Z_2^T M_h^{(r)} Z_2 D_h^{(r)} \sum_{j=1}^{n} \tau_{jh}^{(r)},
\end{aligned}$$

$$
\begin{aligned}
\epsilon_{jh}^{(r)} &= \operatorname{cov}(\epsilon_{jh}|y, \Psi^{(r)}) \\
&= y_j - X_h\beta_h^{(r)} - Z_1 u_{jh}^{(r)} - Z_2 v_h^{(r)}, \\
S_{eh}^{(r)} &= \operatorname{cov}(\epsilon_{jh}|y, \Psi^{(r)}) \\
&= (\sum_{j=1}^{n} \tau_{jh}^{(r)})\Omega_h^{(r)} - \Omega_h^{(r)} M_h^{(r)} \Omega_h^{(r)} \\
&\quad -(\sum_{j=1}^{n} \tau_{jh}^{(r)} - 1)\Omega_h^{(r)} \Sigma_h^{(r)-1} \Omega_h^{(r)}.
\end{aligned}
$$

In addition, we need to calculate the following conditional expectations in order to obtain the Q-function.

$$
\begin{aligned}
E(\epsilon_{jh}^T \Omega_h^{-1} \epsilon_{jh}|y, \Psi^{(r)}) &= \operatorname{trace}(\Omega_h^{(r)-1} E(\epsilon_{jh}\epsilon_{jh}^T|y, \Psi^{(r)})), \\
E(u_{jh}^T A_h^{-1} u_{jh}|y, \Psi^{(r)}) &= \operatorname{trace}(A_h^{(r)-1} E(u_{jh} u_{jh}^T|y, \Psi^{(r)})), \\
E(v_h^T D_h^{-1} v_h|y, \Psi^{(r)}) &= \operatorname{trace}(D_h^{(r)-1} E(v_h v_h^T|y, \Psi^{(r)})),
\end{aligned}
$$

which can be obtained via the relations between the conditional expectations, such as,

$$
E(\epsilon_{jh}\epsilon_{jh}^T) = \operatorname{cov}(\epsilon_{jh}) + E(\epsilon_{jh})E(\epsilon_{jh}^T).
$$

The M-step: Estimation of Parameters

In the M-step of the EM algorithm, we update the estimates that maximize the Q-function with respect to $\Psi^{(r)}$. With the conditional expectations given in the previous section, the updating formulae for $\Psi^{(r+1)}$ are given by

$$
\begin{aligned}
\pi_h^{(r+1)} &= \textstyle\sum_{j=1}^{n} \tau_{jh}^{(r)}/n, \\
\beta_h^{(r+1)} &= \beta_h^{(r)} + G_h^{(r)} \textstyle\sum_{j=1}^{n} \tau_{jh}^{(r)} (y_i - X_h\beta_h^{(r)}) / \textstyle\sum_{j=1}^{n} \tau_{jh}^{(r)}, \\
G_h^{(r)} &= [X_h X_h]^{-1} X_h^T M_h^{(r)} \Omega_h^{(r)}, \\
\Omega_h^{(r+1)} &= \textstyle\sum_{j=1}^{n} \tau_{jh}^{(r)} \epsilon_{jh}^{(r)} \epsilon_{jh}^{(r)T} / \textstyle\sum_{j=1}^{n} \tau_{jh}^{(r)} + S_{eh}^{(r)}, \\
D_h^{(r+1)} &= v_h^{(r)} v_h^{(r)T} + S_{vh}^{(r)}.
\end{aligned}
$$

It is noted that $\Omega_h^{(r+1)}$ and $D_h^{(r+1)}$ may have special structures such as those given in Ng et al. [1]. For the estimation of AR(1) components, after the simplification that is analogous to that for the log-normal survival model with correlated frailty [2], we have

$$\theta_h^{(r+1)} = [(1 + \rho_h^{(r)2})L_{1h} - 2\rho_h^{(r)} L_{2h} - \rho_h^{(r)2} L_{3h}]/(m \sum_{j=1}^{n} \tau_{jh}^{(r)}),$$

where $L_{1h} = \text{trace}(IV_h^{(r)})$, $L_{1h} = \text{trace}(HV_h^{(r)})$, and $L_{1h} = \text{trace}(KV_h^{(r)})$, and where $V_h^{(r)} = \sum_{j=1}^{n} \tau_{jh}^{(r)} (u_{jh}^{(r)} u_{jh}^{(r)T} + S_{uh}^{(r)})$ and I, H and K are m by m matrices, where I is the identity matrix; H has its sub-diagonal entries ones and zeros elsewhere; K takes on the value 1 at the first and last element of its principal diagonal and zeros elsewhere.

Estimation of the correlation parameter ρ_h requires solving the cubic equation,

$$c_{1h}\rho^3 + c_{2h}\rho^2 + c_{3h}\rho + c_{4h} = 0,$$

where $c_{1h} = (m-1)(L_{1h} - L_{3h})$, $c_{2h} = (2-m)L_{2h}$, $c_{3h} = mL_{3h} - (m+1)L_{1h}$ and $c_{4h} = mL_{2h}$. Standard numerical algorithms such as Newton-Raphson may be used to solve for $\rho_h^{(r+1)}$.

Sometimes all components may share common AR(1) parameters, in which case, we just replace $V_h^{(r)}$ with

$$V_h^{(r)} = \sum_{h=1}^{g} \sum_{j=1}^{n} \tau_{jh}^{(r)} (u_{jh}^{(r)} u_{jh}^{(r)T} + S_{uh}^{(r)})$$

in the above approach. The common AR(1) parameters are then obtained using the same formula above.

References

1. Ng SK, McLachlan GJ, Wang K, B T Jones L, Ng SW: **A mixture model with random-effects components for clustering correlated gene-expression profiles**. *Bioinformatics* 2006, **22**:1745–1752.
2. Yau KKW, McGilchrist CA: **ML and REML estimation in survival analysis with time dependent correlated frailty**. *Statistics in Medicine* 1998, **17**:1201–1213.
